# Improved Bioactivity of 3-O-β-D-Glucopyranosyl Platycosides in Biotransformed *Platycodon grandiflorum* Root Extract by Pectinase from *Aspergillus aculeatus*

**DOI:** 10.4014/jmb.2102.02025

**Published:** 2021-04-14

**Authors:** Jung-Hun Ju, Tae-Eui Lee, Jin Lee, Tae-Hun Kim, Kyung-Chul Shin, Deok-Kun Oh

**Affiliations:** Department of Bioscience and Biotechnology, Konkuk University, Seoul 05029, Republic of Korea

**Keywords:** Platycodi radix, 3-O-β-D-glucopyranosyl platycosides, anti-inflammation, antioxidant, tyrosinase inhibition

## Abstract

*Platycodon grandiflorum* (balloon flower) root (Platycodi radix, PR) is used as a health supplement owing to its beneficial bioactive properties. In the present study, the anti-inflammatory, antioxidant, and whitening effects of deglycosylated platycosides (saponins) from PR biotransformed by pectinase from *Aspergillus aculeatus* were investigated. The bioactivities of the platycosides improved when the number of sugar moieties attached to the aglycone platycosides was decreased. The deglycosylated saponins exhibited higher lipoxygenase inhibitory activities (anti-inflammatory activities) than the precursor platycosides and the anti-inflammatory compound baicalein. The 2,2-diphenyl-1-picrylhydrazyl (DPPH) radical scavenging activity of the pectinase-treated PR extract was higher than that of the non-treated PR extract. The trolox-equivalent antioxidant capacity (TEAC) assay showed improved values as the saponins were hydrolyzed. The tyrosinase inhibitory activities (whitening effects) of deglycosylated platycosides were higher than those of the precursor platycosides. Furthermore, 3-O-β-D-glucopyranosyl platycosides showed higher anti-inflammatory, antioxidant, and whitening activities than their precursor glycosylated platycosides. Therefore, 3-O-β-D-glucopyranosyl platycosides may improve the beneficial effects of nutritional supplements and cosmetic products.

## Introduction

*Platycodon grandiflorus* A.DC., known as bellflower or balloon flower, belongs to the genus *Platycodon* of the family Campanulaceae. It is a perennial herb native to East Asia. Platycodi radix (PR), the root of *Platycodon grandiflorum*, contains a mixture of different chemical compounds that may act individually, additively, or synergistically to improve human health. The main bioactive compounds of PR are platycodin saponins. PR is eaten as a side dish in Korean cuisine and is also used in desserts, teas, flavored liquors, and as a dietary supplements for the treatment of pulmonary diseases and respiratory disorders such as cough, asthma, bronchitis, cold, sore throat, tonsillitis, tuberculosis, inflammation, and chest congestion [1]. Furthermore, PR has been used as a traditional herbal treatment for hyperlipidemia, hypertension, and diabetes [2]. The root extract is reported to have hepatoprotective [3], anti-inflammatory [3], anti-lipidemic [4], anti-hypercholesterolemic [5], and anti-obesity properties [6]. PR extract-derived platycosides are composed of pentacyclic triterpenes with two side sugar moieties. One of the sugars is a *β*-glucopyranose residue that is linked by a glycosidic bond at C-3 in the triterpenoid structure, whereas the other is an oligosaccharide (apiofuranosyl-xylopyranosyl-rhamnopranosyl-arabinofuranosyl residue) attached to the ester linkage at C-28.

**Fig. 1:**
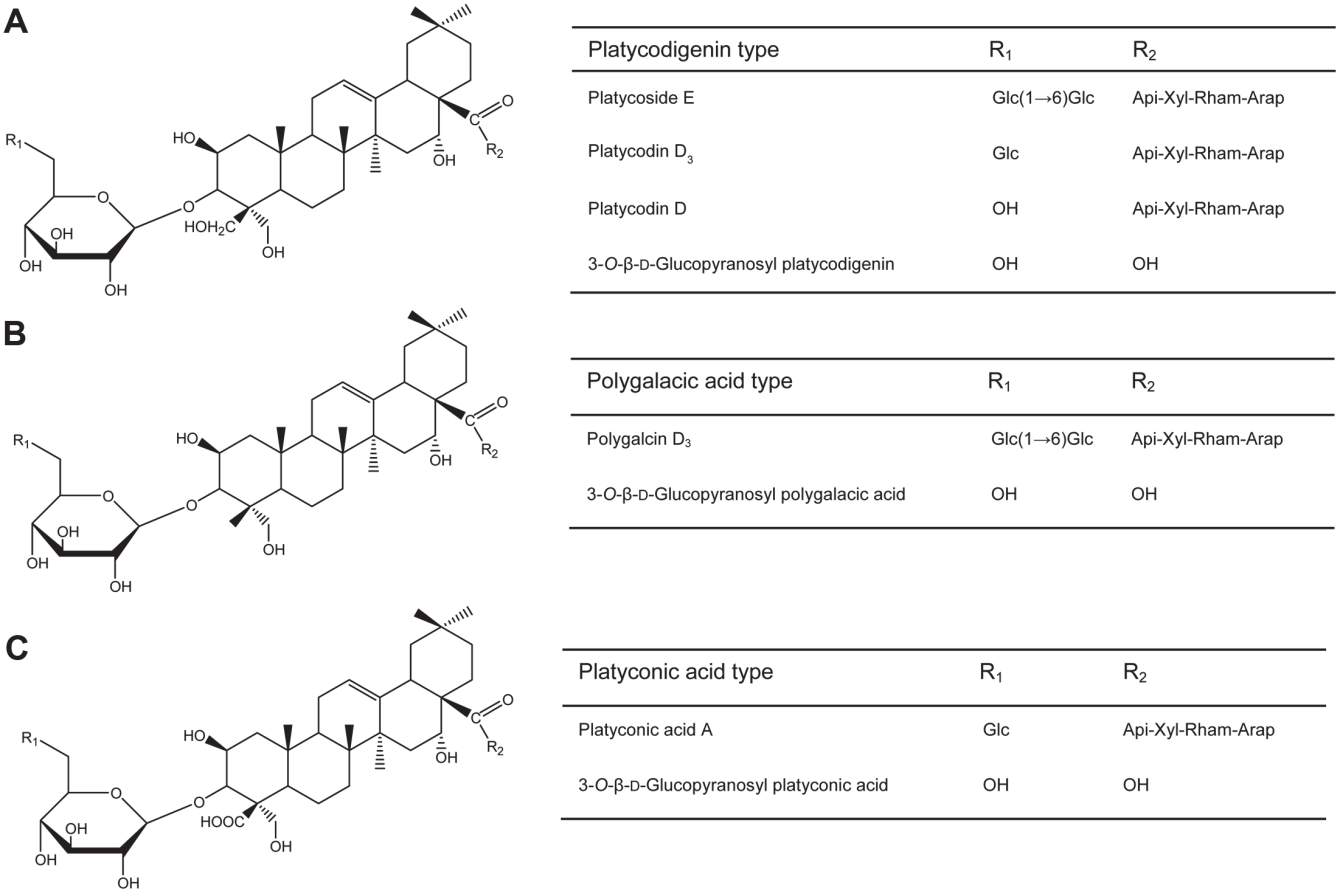
Chemical structures of platycosides used to measure their bioactivities. (**A**) Platycodigenin-type saponin. (**B**) Polygalacic acid-type saponin. (**C**) Platyconic acid-type saponin.

There are three types of saponins, each determined by the type of residue at C-24 in the triterpenoid structure. Platycodigenin-type saponins, one of the three, have a hydroxymethyl group at C-24 (Fig. 1A). Another, polygalacic acid-type saponins, have a methyl group at C-24 (Fig. 1B). The last, platyconic acid-type saponins, have a carboxyl group at C-24 (Fig. 1C). All three types share the same structure except for the residue in C-24, while the differences in functionality depend on the residue of C-24 in the triterpenoid structure.

**Fig. 2:**
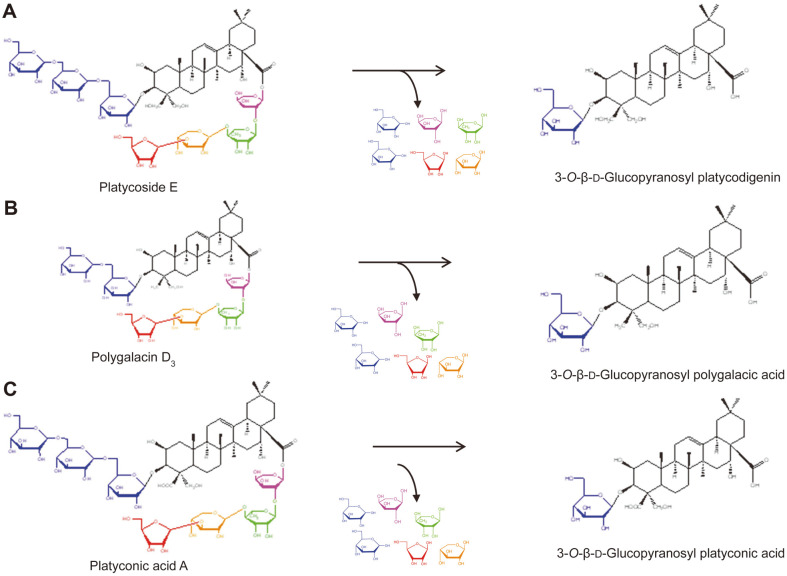
Biotransformation of glycosylated platycosides into 3-O-*β*-D-glucopyranosyl platycosides using pectinase from *A. aculeatus*. (**A**) Biotransformation of platycodigenin-type saponin. PE is converted into 3-O-β-Dglucopyranosyl platycodigenin using pectinase. (**B**) Biotransformation of polygalacic acid-type saponin. Polygalacin D3 is converted into 3-O-β-D-glucopyranosyl polygalacic acid using pectinase. (**C**) Biotransformation of platyconic acid-type saponin. Platyconic acid A is converted into 3-O-β-D-glucopyranosyl platyconic acid using pectinase. Blue, β-D-glucopyranose; pink, α-L-arabinopyranose; green, α-L-rhamnopyranose; orange, β-D-xylopyranose; and red, β-D-apiofuranose.

Deglycosylated saponins, obtained through the biotransformation of glycosylated saponins, exert stronger biological effects than their glycosylated forms [7, 8]. Glycosylated saponins with more than three sugar residues are poorly absorbed in the intestine, whereas deglycosylated saponins, with less than two sugar residues, function as active compounds and are easily absorbed into the bloodstream from the gastrointestinal tract [9]. Glycosylated platycosides are metabolized to deglycosylated platycosides by human intestinal bacteria [10]. The commercial enzymes snailase [11], laminarinase [12], and cellulase [13] also convert deapiosylated platycoside E (deapi-PE) and platycoside E (PE) into deapiosylated platycodin D (deapi-PD) and platycodin D (PD) via deapiosylated platycodin D_3_ (deapi-PD_3_) and platycodin D_3_ (PD_3_), respectively. The β-glucosidase from *Dictyoglomus turgidum* converts PD into deglucosylated PD (deglu-PD) [14], and pectinase from *Aspergillus aculeatus* converts the platycosides in PR into 3-O-β-D-glucopyranosyl platycosides by the hydrolysis of the glucose molecules, leaving a glucose residue at C-3 and the oligosaccharide moiety at C-28 (Fig. 2) [15].

To the best of our knowledge, the pharmacological and nutraceutical activities of deglycosylated platycosides (3-O-β-D-glucopyranosyl platycosides) have not been studied. Therefore, in this study, we investigated the anti-inflammatory, antioxidant, and whitening effects of deglycosylated platycosides by determining lipoxygenase (LOX) inhibitory activity; total antioxidant capacity (TAC) and 2,2-diphenyl-1-picrylhydrazyl (DPPH) radical scavenging activity; and tyrosinase inhibitor activity, respectively. The antioxidant and anti-inflammatory properties of 3-O-β-D-glucopyranosyl platycosides, or extracts containing these platycosides, can be used as functional additives in the health foods and pharmaceutical industries, while the tyrosinase inhibitory effects can be useful in the whitening cosmetics industry. We also compared these properties to those of glycosylated platycosides.

## Materials and Methods

### Materials

*P. grandiflorum* root (PR) was purchased from a local market (Republic of Korea). Platycoside standards, including deapi-PE, PE, deapi-PD, PD_3_, polygalacin D_3_, PD, and platyconic acid A, and pectinase from *Aspergillus aculeatus* as the commercial enzyme (Pectinex Ultra SP-L), were purchased from Ambo Institute (Republic of Korea) and Novozymes (Denmark), respectively. DPPH was obtained from Thermo Fisher Scientific (USA).

### Preparation of 3-O-β-D-Glucopyranosyl Platycosides and Other Platycosides

To prepare 3-O-β-D-glucopyranosyl platycodigenin, 3-O-β-D-glucopyranosyl polygalacic acid, and 3-O-β-D-glucopyranosyl platyconic acid standards, the 3-O-β-D-glucopyranosyl platycoside product solutions were obtained from the reactions at 50°C with pectinase from *A. aculeatus* in 50 mM citrate-phosphate buffer (pH 5.0) containing 10 mg/ml enzyme and 1 mg/ml of reagent-grade PE, polygalacin D_3_, and platyconic acid A (PA) as substrates, respectively, after 24 h. PR extract and 3-O-β-D-glucopyranosyl platycoside product solutions were separated with preparative high-performance liquid chromatography (Prep-HPLC) (Agilent 1260, USA) equipped with a Hydrosphere C18 prep column (10 × 250 mm, 5 μm particle size; YMC, Japan), eluted with water at 30°C at a flow rate of 4.7 ml/min. The absorbance of the eluent was monitored at 203 nm and collected using a fraction collector. The peak area ratios of PA, polygalacin D, 3′′-O-acetyl polygalacin D_3_, and deapi-PD3 obtained from the purification of glycosylated platycosides from PR extract to total area in HPLC chromatograms were approximately 90%. The 3-O-β-D-glucopyranosyl platycosides showed 98% purity, as calculated from the ratio of the molar amount obtained after the purification of the products to the molar amount of the substrates.

### Preparation of PR Extract and Biotransformed Extract

The dried root of *P. grandiflorum* (100 g) was suspended in 1 L of 99.8% (v/v) methanol and incubated at 80°C for 24 h [16, 17]. After incubation, the precipitates were eliminated with vacuum filtration through a filter with a pore size of 0.45 μm. The methanol was removed by evaporation and the dried residue was dissolved in 1 L of water. The methanol-free solution was applied to a column containing Diaion HP-20 resin (500 mm × 12 mm). Other hydrophilic compounds and free sugars were removed by washing the column with water, and the adsorbed platycosides in the resin were extracted by sequentially eluting with 2 L of methanol at a flow rate of 0.5 ml/min. The methanol in the extracted platycosides was removed by evaporation and the dried residue was dissolved in 1 L of water. The dissolved solution was diluted to 7.4% (w/v) PR extract by adjusting the concentration of PE to 1.0 mg/ml, which was used as PR extract. The biotransformation of PR extract was performed at 50°C in 50 mM citrate-phosphate buffer (pH 5.0) containing 10 mg/ml pectinase and 7.4% (w/v) PR extract containing 1 mg/ml PE, 0.05 mg/ml polygalacin D_3_, and 0.17 mg/ml platyconic acid A for 36 h. After the biotransformation, the reaction solution was used as biotransformed extract.

### LOX Inhibitory Assay

The LOX inhibitory activity assay to evaluate anti-inflammatory activity was conducted using 4 μM of each sample and a LOX Inhibitor Screening Assay Kit (Cayman Chemical, USA). The mole concentration of PR extract was 2,051 μM because it contained 49 μM deapi-PE, 645 μM PE, 8 μM deapi-PD_3_, 29 μM PD_3_, 36 μM polygalacin D_3_, 18 μM deapi-PD, 220 μM PD, 662 μM polygalacin D, 113 μM 3′′-O-acetyl polygalacin D_3_, 134 μM platycodin A, and 137 μM platyconic acid A, and the mole concentration of biotransformed extract was 1,353 μM because it contained 894 μM 3-O-β-D-glucopyranosyl platycodigenin, 315 μM 3-O-β-D-glucopyranosyl polygalacic acid, and 144 μM 3-O-β-D-glucopyranosyl platyconic acid [15]. PR extract and biotransformed PR extract were diluted to 4 μM to test anti-inflammatory activity.

The positive controls were nordihydroguaiaretic acid (NDGA) and baicalein (5,6,7-trihydroxyflavone), which served as standard LOX inhibitory and anti-inflammatory compounds, respectively. First, 15-LOX (90 μl) and the samples (10 μl) were loaded into individual wells of 96-well plates. The reaction was initiated by adding arachidonic acid (10 μl), a substrate for LOX, to each well and the plate was incubated on a shaker for 5 min. Next, chromogen (100 μl) was added to each well to terminate the reaction. The amount of hydroperoxide formed from arachidonic acid by the action of 15-LOX was determined using UV spectroscopy by measuring the absorbance at 500 nm. LOX inhibitory activity (%) was calculated as follows: (*C−T*)/C × 100, where *C* and *T* represent the enzyme activity without and with the test samples, respectively. This calculation was referred to the kit protocol.

### TAC Assay

To determine the antioxidant activity, the total antioxidant capacity was measured using a TAC Assay Kit (Dogenbio, Republic of Korea). Ascorbic acid (Sigma Aldrich, USA) was used as a positive control. A standard curve was constructed by relating the absorbances at 450 nm and the concentrations of 6-hydroxy-2,5,7,8-tetramethylchroman-2-carboxylic acid (trolox) at 0.1, 0.3, 0.5, 1, and 2 mM. Copper reagent (100 μl) and reaction buffer (100 μl) were added to each sample or standard (100 μl). Copper reagent (100 μl) and ethanol (100 μl) were added to the blank (100 μl). After the reactions had progressed for 30 min at room temperature, 120 μl of each mixture was loaded into each well of a 96-well plate and the absorbance was measured at 450 nm using a microplate reader (BioTek, USA). The absorbance of the blank was deducted from that of the samples. The absorbance of each sample was converted into the concentration of trolox using the standard curve.

### DPPH Radical Scavenging Assay

DPPH solution in methanol was prepared at a concentration of 0.2 mM. The PR extract and its biotransformed extract were prepared at concentrations of 0.25, 0.5, 1, and 2 mg/ml. DPPH solution (150 μl) and prepared samples (50 μl) were added into individual wells of a 96-well plate. Methanol (50 μl) was used as corresponding blank. The samples were diluted 4-fold leading to final concentrations of 62.5, 125, 250, and 500 μg/ml. The reactions were performed at 25°C in a 96-well plate for 30 min, and the absorbance at 520 nm was measured using a microplate reader. Radical scavenging activity was calculated using the following equation: (*A_c_* – *A_s_*)/*A_c_* × 100, where *A_c_* was the absorbance of DPPH solution (150 μl) mixed with methanol (50 μl) as a control and As was the absorbance of DPPH solution (150 μl) mixed with the samples (50 μl). Ascorbic acid dissolved in water was used as a positive control.

### Tyrosinase Inhibitor Assay

The tyrosinase inhibitor assay was performed using a Tyrosinase Inhibitor Screening Kit (Bio Vision, USA). Kojic acid was used as a positive control. Samples were dissolved in methanol and tested at a concentration of 0.75 mM. After platycoside samples or kojic acid (20 μl) were added into the individual wells of a 96-well plate, they were each supplemented with tyrosinase enzyme solution (50 μl) and tyrosinase substrate solution (30 μl). The plate was incubated at 25°C for 10 min. The absorbances of all samples were measured at 510 nm for 30−60 min, and the absorbances *Abs*_1_ and *Abs*_2_ were measured at two selected time points within the linear range. All sample slopes, including those of the enzyme activity control (*EC*), were calculated by dividing the net Δ*Abs* (*Abs*_2_−*Abs*_1_) value by time, Δ*T* (*T*_2_−*T*_1_). The relative tyrosinase inhibition (%) was calculated using the following formula: (slope of *EC*−slope of S)/slope of *EC* × 100, where *S* represents the test inhibitor.

## Results and Discussion

### Platycoside Content in PR Extract Before and After Biotransformation by Pectinase from *A. aculeatus*

The platycodigenin-type platycosides deapi-PE, PE, deapi-PD_3_, PD_3_, deapi-PD, PD, and PA; polygalacic acid-type platycosides polygalacin D_3_, polygalacin D, and 3′′-O-acetyl polygalacin D3; and the platyconic acid-type platycoside platyconic acid A in Platycodi radix extract were converted into 3-O-β-D-glucopyranosyl platycodigenin, 3-O-β-D-glucopyranosyl polygalacic acid, and 3-O-β-D-glucopyranosyl platyconic acid, respectively, by pectinase from *A. aculeatus* [15]. The contents of deapi-PE in 7.4% (w/v) PR extract was 2.53%, and the contents of PE, polygalacin D, and PD as the main compounds were 36.23%, 28.98%, and 9.78%, respectively. After a reaction time of 36 h, the contents of 3-O-β-D-glucopyranosyl platycodigenin, 3-O-β-D-glucopyranosyl polygalacic acid, and 3-O-β-D-glucopyranosyl platyconic acid were 44.2%, 15.2%, and 7.2% (w/w), respectively (Table 1).

### Anti-Inflammatory Activities of 3-O-β-D-Glucopyranosyl Platycosides and Platycosides from PR Extract

**Fig. 3:**
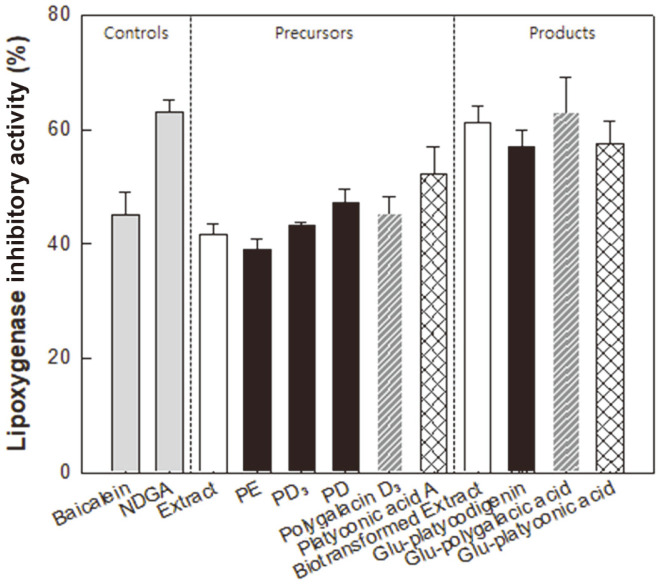
LOX inhibitory activities of PR extract; PE, PD_3_, and PD; polygalacin D3; and platyconic acid A as precursors and biotransformed Platycodi radix extract, 3-O-β-D-glucopyranosyl platycodigenin, 3-O-β-Dglucopyranosyl (Glu-) polygalacic acid, and 3-O-β-D-glucopyranosyl platyconic acid as products. NDGA and baicalein were used as positive controls, which are LOX inhibitory and anti-inflammatory compounds, respectively. Sample and control concentrations were 4 μM. Gray, white, black, hatched, and grid bars represent control, PR extract and the biotransformed PR extract, platycodigenin-type platycosides, polygalacic acid-type platycosides, and platyconic acid-type platycosides, respectively. Data represent means of three experiments and error bars represent standard deviations.

The LOX inhibitory activity assay has been used to assess the anti-inflammatory activity [18]. The LOX inhibitory activities of platycosides and control samples were measured at 4 μM (Fig. 3). The LOX inhibitory activities of the baicalein and NDGA controls were 45% and 63%, respectively. The LOX inhibitory activities of glycosylated platycosides, including the platycodigenin-type platycosides PE, PD_3_, and PD; the polygalacic acid-type platycoside polygalacin D_3_; and the platyconic acid-type platyconic acid A as precursors were 39%, 44%, and 47%; 46%; and 52%, respectively. These platycosides were converted into 3-O-β-D-glucopyranosyl platycodigenin, 3-O-β-D-glucopyranosyl polygalacic acid, and 3-O-β-D-glucopyranosyl platyconic acid, respectively, using pectinase from *A. aculeatus*. After the biotransformation, the LOX inhibitory activities increased to 57%, 63%, and 58%, respectively, indicating that the anti-inflammatory activities of the biotransformed deglycosylated products were higher than those of the precursors. However, the statistical difference between platyconic acid A and 3-O-β-D-glucopyranosyl platyconic acid was not significantly shown. The LOX inhibitory activity of PR extract was 42% and it was increased to 61% after biotransformation, indicating that deglycosylation of saponins by pectinase increased anti-inflammatory activity.

LOXs catalyze the insertion of oxygen into polyunsaturated fatty acids such as arachidonic acid [18]. These LOX-catalyzed oxygenated arachidonic acid products mediate inflammation, and thus LOX inhibition could be used to indicate anti-inflammatory activity. In this study, we evaluated the *in vitro* LOX inhibitory activities of platycosides. The LOX inhibitory activities of the biotransformed PR extract and 3-O-β-D-glucopyranosyl platycosides were higher than that of the positive control anti-inflammatory agent, baicalein (Fig. 3). Moreover, the LOX inhibitory activities of 3-O-β-D-glucopyranosyl platycosides were higher than that of the platycodigenin-type platycoside deglu-PD, obtained from PD through the treatment of *D. turgidum* β-glucosidase [14]. The PD derivatives have been reported to exhibit anti-inflammatory effects [19] and inhibit the production of inflammatory mediators such as nitric oxide and prostaglandin E_2_. However, the anti-inflammatory activities of polygalacic acid-type platycosides and platyconic acid-type platycosides have not yet been reported. In this study, we determined the anti-inflammatory activities of the three platycosides present in PR extract, including the platycodigenin-, polygalacic acid-, and platyconic acid-type platycosides. Among the tested platycosides, 3-O-β-D-glucopyranosyl polygalacic acid exhibited the highest anti-inflammatory activity.

### Antioxidant Activities of PR Extract, Biotransformed PR Extract, Glycosylated Platycosides, and 3-O-β-D-Glucopyranosyl Platycosides

**Fig. 4:**
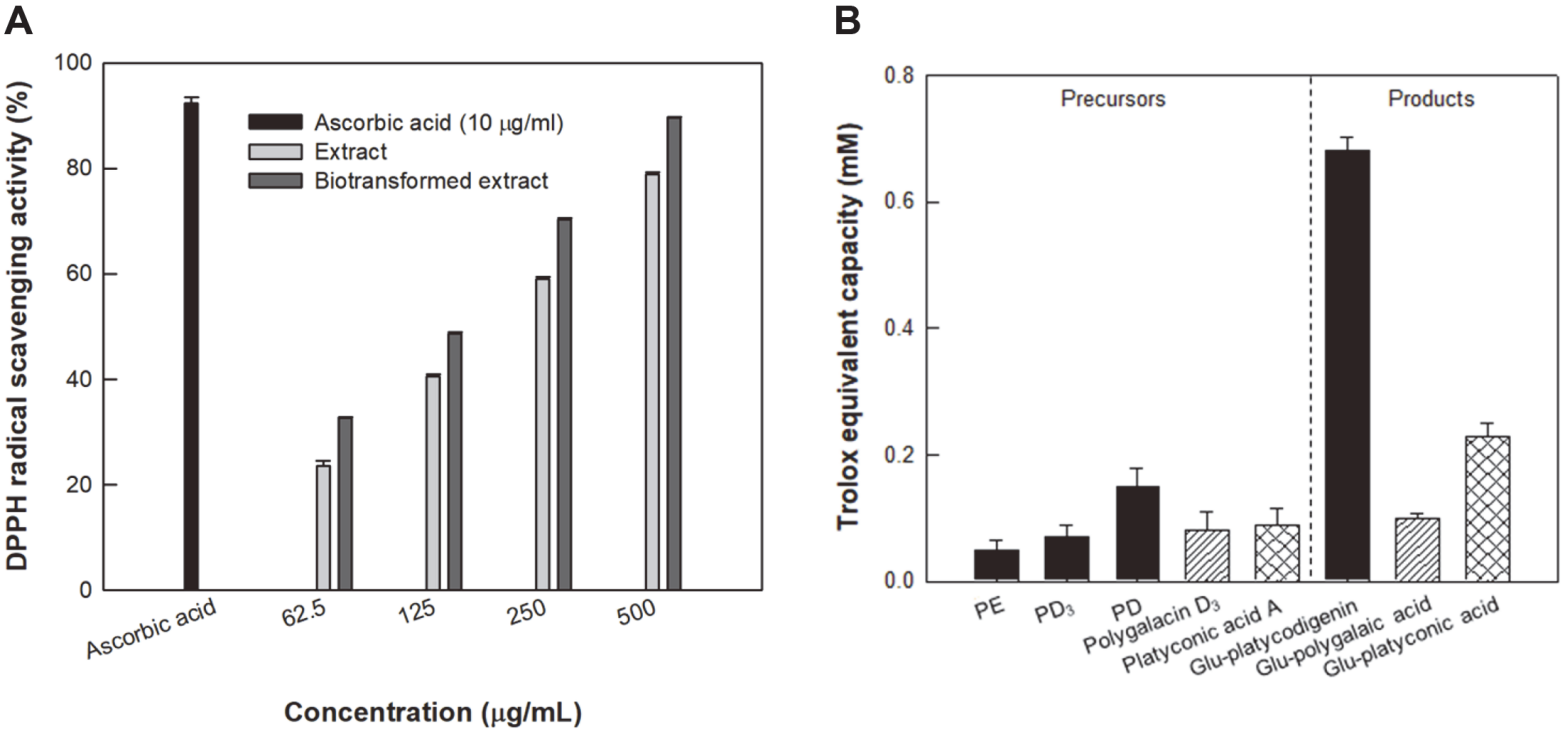
Antioxidant activities of PR extract and platycosides in the PR extract. (**A**) DPPH radical scavenging activities of PR extract and biotransformed PR extract. Ascorbic acid was used as a standard control. Sample concentrations were 62.5, 125, 250, and 500 μg/ml. Black, gray, and dark gray bars represent the control, PR extract, and biotransformed PR extract, respectively. (**B**) TAC activities of PE, PD_3_, and PD; polygalacin D3; and platyconic acid A as precursors and 3-O-β-Dglucopyranosyl platycodigenin, 3-O-β-D-glucopyranosyl polygalacic acid, and 3-O-β-D-glucopyranosyl platyconic acid as products, respectively. Sample and control concentrations were 1 mM. Gray, black, hatched, and grid bars represent control, platycodigenin-type platycosides, polygalacic acid-type platycosides, and platyconic acid-type platycosides, respectively. Data represent means of three experiments and error bars represent standard deviations.

The antioxidant activity was measured using the DPPH assay. The DPPH radical scavenging activities of PR extract, pectinase-treated biotransformed PR extract, and ascorbic acid increased as the corresponding sample concentrations increased from 62.5 to 500 μg/ml (Fig. 4A). The free radical scavenging activity of the positive control ascorbic was 92.4% at 10 μg/ml. The free radical scavenging activities of PR extract and biotransformed PR radix extract increased from 17.5% and 24.3% at 62.5 μg/ml to 76.2% and 86.5% at 500 μg/ml, respectively. These results indicated that the free radical scavenging activity of biotransformed PR extract was higher than that of PR extract. Thus, deglycosylation by pectinase increased antioxidant activity.

The DPPH assay is widely used for testing the radical scavenging ability of components in food samples. We found that the DPPH radical scavenging activity of the biotransformed PR extract was higher than that of the PR extract (Fig. 4A). The DPPH free radical scavenging activity was normalized and expressed as *EC*_50_ (μg/ml), representing the effective concentration of each sample required to show 50% antioxidant activity. The *EC*_50_ values of the PR extract and biotransformed PR extract were 221 ± 14 μg/ml and 184 ± 12 μg/ml, respectively. The *EC*_50_ value of lower biotransformed PR extract indicated better free radical scavenging activity. The hydrolysis of glycoside moieties of the glycosylated platycosides in PR by pectinase-treated deglycosylation resulted in an improvement of antioxidant activity. Thus, the biotransformed PR extract with its improved antioxidant activity could be used as a food supplement. The DPPH free radical scavenging activities have been used to determine the antioxidant activities in the extracts of natural products, but not in single saponins. Thus, the antioxidant activities of single saponins should be determined using a different antioxidant assay.

The antioxidant activity, or total antioxidant capacity (TAC), was expressed as a trolox equivalent capacity (TEC) value of each platycoside, increased by decreasing the number of glycoside residues (Fig. 4B). For example, the antioxidant activities of platycodigenin-type platycosides followed the order 3-O-β-D-glucopyranosyl platycodigenin (one glycoside) > PD (four glycosides) > PD_3_ (six glycosides) > PE (seven glycosides). The TEC values of all 3-O-β-D-glucopyranosyl platycosides improved compared to their precursors. The TEC values of 3-O-β-D-glucopyranosyl platycosides followed the order platycodigenin-type platycosides > platyconic acid-type platycosides > polygalacic acid-type platycosides.

The antioxidant activities of the single saponins, platycodigenin, polygalacic acid, deapi-PE, PD, and PE have been investigated using a total oxidant-scavenging capacity (TOSC) assay [20]. The antioxidant capacities of these platycosides increased as the C-3 glucose and C-28 glycoside residues of the platycodigenin-type platycosides were hydrolyzed. Aglycone platycodigenin, a saponin without glycoside residues, showed the highest antioxidant activity. However, aglycone polygalacic acid did not exhibit antioxidant activity. We used the TAC assay as an alternative assay for determining the antioxidant activities of single saponins. In this assay, trolox was used as a standard antioxidant, and other compounds were converted to trolox-equivalents in order to assess their antioxidant capacities. Ascorbic acid was used as a positive control. The antioxidant activities of 3-O-β-D-glucopyranosyl platycosides were measured for the first time and the activity of 3-O-β-D-glucopyranosyl platycodigenin was found to be the highest among the platycosides (Fig. 4B), pointing to 3-O-β-D-glucopyranosyl platycodigenin as an effective antioxidant. The difference in the structures of the three platycosides is that they have different residues at C-24. The platycodigenin, polygalacic acid, and platyconic acid types had hydroxymethyl, methyl, and carboxyl residues, respectively. The strongest antioxidant activity of 3-O-β-D-glucopyranosyl platycodigenin may be due to the hydroxymethyl residue.

### Whitening Activities of Glycosylated Platycosides and 3-O-β-D-Glucopyranosyl Platycosides

**Fig. 5:**
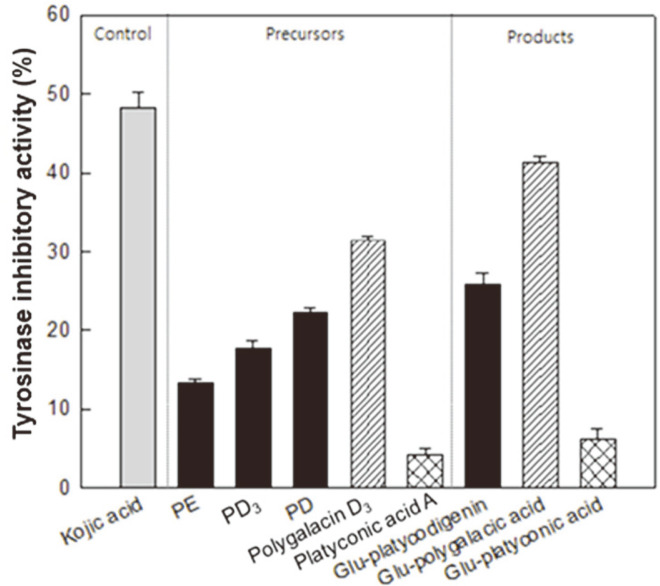
Tyrosinase inhibition activities of PE, PD_3_, and PD, polygalacin D_3_, and platyconic acid A as precursors and 3-O-β-D-glucopyranosyl platycodigenin, 3-O-β-D-glucopyranosyl polygalacic acid, and 3- O-β-D-glucopyranosyl platyconic acid as products, respectively. Sample and control concentrations were 0.75 mM. Kojic acid, a whitening agent, was used as a control. Gray, black, hatched, and grid bars represent control, platycodigenin-type platycosides, polygalacic acid-type platycosides, and platyconic acid-type platycosides, respectively. Data represent means of five experiments and error bars represent standard deviation.

We tested the tyrosinase inhibitory effect of glycosylated platycosides and 3-O-β-D-glucopyranosyl platycosides. The relative tyrosinase inhibition of kojic acid, a commonly used control in tyrosinase inhibition experiments, was 49%. Glycosylated platycosides, including the platycodigenin-type platycosides PE, PD_3_, and PD, the polygalacic acid-type platycoside polygalacin D_3_, and the platyconic acid-type platycoside platyconic acid A as precursors exhibited 12%, 17%, and 23%; 30%; and 4% relative tyrosinase inhibition, respectively, while the platycosides 3-O-β-D-glucopyranosyl platycodigenin, 3-O-β-D-glucopyranosyl polygalacic acid, and 3-O-β-D-glucopyranosyl platyconic acid showed 28%, 39%, and 7% relative tyrosinase inhibition, respectively (Fig. 5). As a result, the relative tyrosinase inhibitory effect in platycosides improved as the number of sugars attached to aglycone platycosides decreased.

PD is a whitening agent because it is effective in inhibiting melanogenesis via suppressing the cAMP signaling pathway [21]. Tyrosinase inhibition is effective in whitening as it is involved in melanin synthesis. We found that the tyrosinase inhibitory effect of platycosides improved as the platycoside-linked sugars were hydrolyzed (Fig. 5). The polygalacic acid-type platycosides showed higher tyrosinase inhibitory activities than those of the platycodigenin-type platycosides and platyconic acid-type platycosides. The whitening effect of 3-O-β-D-glucopyranosyl polygalacic acid was higher than that of the whitening agent PD [21]. Thus, 3-O-β-D-glucopyranosyl polygalacic acid could be an effective whitening agent.

In summary, the deglycosylated platycosides, 3-O-β-D-glucopyranosyl platycosides, displayed increased anti-inflammatory, antioxidant, and whitening activities compared to glycosylated platycosides. To the best of our knowledge, this is the first report on the bioactivities of 3-O-β-D-glucopyranosyl platycosides. The anti-inflammatory activities of 3-O-β-D-glucopyranosyl platycosides were higher than that of the anti-inflammatory agent baicalein. The antioxidant activity improved as the saponins were hydrolyzed, and the activity of 3-O-β-D-glucopyranosyl platycodigenin was the highest among the platycosides. The whitening activity of 3-O-β-D-glucopyranosyl polygalacic acid was higher than the previously used whitening platycoside PD, respectively. Thus, 3-O-β-D-glucopyranosyl platycosides might be useful as food supplements or in the use of cosmetic formulations.

## Figures and Tables

**Table 1:** Contents of platycosides in Platycodi radix (PR) extract before and after biotransformation by pectinase from *Aspergillus aculeatus*.

Platycoside	Before biotransformation		After biotransformation	

	Content (%, w/w)	Concentration (mg/ml)	Content (%, w/w)	Concentration (mg/ml)
Deapi-platycoside E	2.53	0.07	0	0
Ddeapi-platycodin D_3_	0.36	0.01	0	0
Deapi-platycodin D	0.72	0.02	7.97	0.11
Platycoside E	36.23	1.00	0	0
Platycodin D_3_	1.45	0.04	7.24	0.10
Platycodin D	9.78	0.27	18.1	0.25
Platycodin A	6.15	0.17	0	0
Polygalacin D_3_	1.81	0.05	0	0
Polygalacin D	28.98	0.80	0	0
3″-*O*-Acetyl polygalacin D_3_	5.80	0.16	0	0
Platyconic acid A	6.15	0.17	0	0
3-*O*-β-d-Glucopyranosyl platycodigenin	ND	ND	44.20	0.61
3-*O*-β-d-Glucopyranosyl polygalacic acid	ND	ND	15.22	0.21
3-*O*-β-d-Glucopyranosyl platyconic acid	ND	ND	7.24	0.10
Total	100	2.76	100	1.38

ND: not detected.

The concentration of Platycodi radix extract was 7.4% (w/v).
